# Individual, social, environmental, and physical environmental correlates with physical activity among Canadians: a cross-sectional study

**DOI:** 10.1186/1471-2458-9-21

**Published:** 2009-01-16

**Authors:** Sai Yi Pan, Christine Cameron, Marie DesMeules, Howard Morrison, Cora Lynn Craig, XiaoHong Jiang

**Affiliations:** 1 Evidence and Risk Assessment Division, Centre for Chronic Disease Prevention and Control, Public Health Agency of Canada, Ottawa, Canada; 2 Canadian Fitness and Lifestyle Research Institute, Ottawa, Canada; 3 Science Office, Centre for Chronic Disease Prevention and Control, Public Health Agency of Canada, Ottawa, Canada; 4 Faculty of Applied Health Sciences, University of Waterloo, Waterloo, Canada

## Abstract

**Background:**

The identification of various individual, social and physical environmental factors affecting physical activity (PA) behavior in Canada can help in the development of more tailored intervention strategies for promoting higher PA levels in Canada. This study examined the influences of various individual, social and physical environmental factors on PA participation by gender, age and socioeconomic status, using data from the 2002 nationwide survey of the Physical Activity Monitor.

**Methods:**

In 2002, 5,167 Canadians aged 15–79 years, selected by random-digit dialling from household-based telephone exchanges, completed a telephone survey. The short version of the International Physical Activity Questionnaire was used to collect information on total physical activity. The effects of socio-economical status, self-rated health, self-efficacy, intention, perceived barriers to PA, health benefits of PA, social support, and facility availability on PA level were examined by multiple logistic regression analyses.

**Results:**

Self-efficacy and intention were the strongest correlates and had the greatest effect on PA. Family income, self-rated health and perceived barriers were also consistently associated with PA. The effects of the perceived health benefits, education and family income were more salient to older people, whereas the influence of education was more important to women and the influence of perceived barriers was more salient to women and younger people. Facility availability was more strongly associated with PA among people with a university degree than among people with a lower education level. However, social support was not significantly related to PA in any subgroup.

**Conclusion:**

This study suggests that PA promotion strategies should be tailored to enhance people's confidence to engage in PA, motivate people to be more active, educate people on PA's health benefits and reduce barriers, as well as target different factors for men and women and for differing socio-economic and demographic groups.

## Background

Regular physical activity (PA) is an important part of a healthy lifestyle. Considerable evidence shows that regular PA has many physical and mental health benefits, such as reduction in all-cause mortality and prevention of cardiovascular diseases, type II diabetes, hypertension, several types of cancers, osteoporosis, anxiety, and depression [1,2]. In seniors, regular PA has additional benefits, including increasing longevity, reducing pain from arthritis, decreasing risks of falls and fractures, and increasing ability to maintain functional independence [1]. This may be particularly important to those countries with an aging population.

Because of the multiple health benefits of regular PA, many health organizations have recommended 30 minutes or more of moderate-intensity PA at least 4 or 5 days a week [2,3]. Despite the creation of such public health recommendations, many people do not participate regularly in PA [4].

Whether people adopt an active lifestyle is a complex behavioural process that is influenced by various factors [5-7]. Social-ecological models propose that health behaviours (e.g., PA) are influenced by the interplay of multiple levels of factors (personal, social and institutional environmental, and physical environmental) and emphasize the need to address variables at multiple levels to understand and change health behaviours [8]. Personal factors include biological (e.g., age, gender and health status), psychosocial (e.g., intention, self-efficacy, health beliefs about PA and perceived barriers to PA), and others (e.g., education). Studies have identified positive associations of PA with intention, self-efficacy, perceived benefits of PA and good health status, but negative associations with perceived barriers to PA, older age and being female [5]. Social support and social networks, the characteristics of the social-environment, such as companionship, encouragement, assistance from friends/family members/others, tangible aid and service from community, and advice, suggestions and information from professionals, have all been shown to have positive influences on PA; while social inequality including income inequality and racial discrimination may have negative impacts on PA behaviour [6]. Other dimensions of the social environment such as social cohesion, social capital and neighbourhood SES are also related to PA behaviour [6]. Supportive physical environments, both perceived and objectively measured, were also associated with higher PA level [7-10]. Some examples of the supportive physical environments are: available, accessible and convenient PA facilities, presence of sidewalks and bike paths, safe streets, good lighting of streets, aesthetics, and good urban design (high density, greater connectivity, mixed land use, and inclusion of walking/bike paths and green spaces in community development, etc.).

People in various demographic subgroups may differ according to the determinants of PA. Research has shown different patterns of correlates for men and women [11-13], for different age groups [13], and for socioeconomic status [12,14]. For example, social support for PA and lack of time may be more influential for women [11], and there are differential impacts of perceived PA benefits on PA participation for men and women, and for old and young people [13].

Social-ecological models recognize the need to address factors at multiple levels in order to understand and change PA behaviours, and multilevel approaches derived from these models have been recommended to examine PA determinants [8]. Although this approach has been used in the US, Australia and other countries, it has rarely been applied in Canada. Examination of the potential influence of these factors in a Canadian context would help in developing more tailored intervention strategies for promoting higher PA level in Canada. Therefore, we used a nationwide survey to identify factors that are associated with PA participation in a Canadian population. The research questions were as follows:

• Do individual factors, social support and physical environment have independent effects on PA in the Canadian population?

• Do the influences of these variables on PA vary by gender, age, education level and family income level?

## Methods

This study used data from the 2002 Physical Activity Monitor. It was the ninth nationwide survey on PA conducted by the Canadian Fitness and Lifestyle Research Institute (CFLRI) after the 1981 Canada Fitness Survey, the 1988 Campbell Survey on Well-Being in Canada, and the 1995, 1997, 1998, 1999, 2000 and 2001 waves of the Physical Activity Monitor.

### Survey design

The participants were selected using random-digit dialling from household-based telephone exchanges. The random sample of households was selected roughly proportional to the population in each province and territory with a minimum of 250 adults for each jurisdiction. Within each selected household, one individual over the age of 15 was chosen at random, thus providing a random sample of 5,303 individuals in Canada. Data from the Physical Activity Monitor was collected throughout the full calendar year of 2002. The data were captured directly during the interviews using a computer-assisted telephone interview system. The overall response rate was 51%. A total of 5,167 participants aged 15–79 years were used for this analysis.

### Measures

The social-ecological model was used as the framework for the survey. The content of the 2002 Physical Activity Monitor was determined by the CFLRI, in collaboration with the Physical Activity Unit of the Public Health Agency of Canada, and the provincial and territorial government departments concerned with fitness, active living, leisure, sport and recreation through the auspices of the Interprovincial Sport and Recreation Council. The participants were asked about their socio-demographic background information (age, marital status, employment status, education, household income and gender), self-rated health, PA patterns, attitudes, and awareness of PA opportunities.

### Physical activity measures

The PA questionnaire used in this survey is the short form version of the International Physical Activity Questionnaire (IPAQ), which has been shown previously to have an acceptable test-retest reliability and criterion validity [15]. Participants were asked the number of days they did vigorous PA, moderate PA (not including walking) and walking, as well as the number of hours and minutes per day they did the three kinds of activities in the last 7 days respectively. The physical activity included any PA that people did: 1) at work, 2) as part of house and yard work, 3) to get from place to place, and 4) in spare time for recreation, exercise or sport, but with no information on frequency and duration for these separate domains. We calculated respective total hours for vigorous PA, moderate PA and walking. A MET-hours was derived by multiplying the respective total hours with the metabolic equivalent (MET) value of vigorous PA (MET = 8.0), moderate PA (MET = 4.0) and walking (MET = 3.3), and then adding all three . The physical activity index, Sufficient PA, was defined as at least 3 days of vigorous activity of at least 20 minutes per day; OR 7 days of moderate-intensity activity and/or walking of at least 30 minutes per day; OR 7 days of any combination of walking, moderate-intensity or vigorous-intensity activities achieving a minimum of 840 MET-minutes/week.

### Individual-level variables

#### Intention

Participants were asked: "To what extent do you intend to be physically active over the next six months?" rated via a 7-point Likert scale from 1 (no intention at all) to 7 (fully intend to be physically active).

#### Perceived health benefits of and barriers to physical activity

The perceived health benefits regarding PA were assessed by 4 items. Using a 7-point Likert scale from 1 to 7, where 1 means do not agree and 7 means agree very strongly, participants were asked: "To what extent do you agree with the following statement?": regular PA helps prevent heart disease, prevent cancer, reduce stress, and maintain the ability to do every day tasks in older age. Perceived personal barriers to PA were assessed using 8 items using a 7-point Likert scale, ranging from 1 (not at all important) to 7 (very important). Participants were asked: "How important are each of the following in keeping you from participating regularly in physical activity?": lack of time; lack of energy (/too tired); lack of physical skills; lack of interest or motivation; feeling uncomfortable or ill at ease; long-term illness, disability, injury; fear of being injured; and costs. The internal consistency of the scale was 0.72 for perceived health benefits of PA and 0.81 for perceived barriers to PA.

#### Self-efficacy

Participants were asked how confident they were that they could regularly do a total of 30 minutes of moderate PA three or four times a week and a total of 60 minutes of light PA each day, using scales where 1 means not at all confident and 7 means very confident. The internal consistency of this scale was 0.74.

### Social environment variables: Social support

Social support such as instrumental support (tangible aid and service) and informational support (advice, suggestions and information) that influences people to engage in PA was assessed by 8 items (information on PA, health and well beings; help in planning daily schedule; professional help in choosing best types of activities; specific instruction or coaching in different activities; convenient public transportation; affordable facilities, services and programs; affordable support services such as child care, parking; affordable services to link with other people). Participants were asked how important each of the 8 items would be in making it easier for them to be physically active. The items were presented via a 7-point Likert scale from 1 (not important at all) to 7 (very important). The internal consistence of this scale was 0.86.

### Physical environment variables: Facility availability

The availability of PA facilities in respondent's community (PA facilities and programs offered locally in respondent's community) was appraised by 5 items concerning the number of places to safely walk (including sidewalks, walking trails and so on), number of places to safely ride a bike (such as designated bike lanes or special paths), number of publicly owned multi-purpose recreation trails, number of facilities, places and programs that are designed specifically for doing PA and sports (including fitness centres, pools, arenas, tennis or racquet ball courts, etc), and number of other places that could be used for PA (such as school gym used after hours or public places where kids can skateboard). Participants were asked about how many of these 5 types of infrastructure there are in their local communities. The response options were: none at all, some and many.

All of the above scales were developed by the Canadian Fitness and Lifestyle Research Institute for the purpose of the national surveys of the Physical Activity Monitor.

### Statistical analysis

For perceived health benefits, perceived barriers, self-efficacy, social support and facility availability, a total score for each factor was computed by adding the responses on all items for each factor and then a mean score was obtained by dividing the total score by the number of items for each factor. The mean scores of these 5 factors were used in logistic regression analyses.

We used logistic regressions to examine the relationship of PA participation with various socio-demographic factors, self-rated health, intention, perceived health benefits, perceived barriers, self-efficacy, social support and facility availability. When examining these relationships, we took into account the simultaneous effects of other independent variables. The final multivariate models included all variables that showed a statistically significant unadjusted association (*p *< 0.05) with PA. Because education level and family income level were highly correlated and there were 15% of records with a missing value for the family income variable, models were not adjusted for family income level. Age was entered in the models as a continuous variable, gender as a dichotomous variable, self-rated health as an ordinal variable (excellent, good, fair and poor), and education as an ordinal variable (elementary, secondary, college, and university including undergraduate and graduate). Perceived health benefits, perceived barriers, self-efficacy, and facility availability were entered into the model as continuous variables. Because the interaction effects of gender, age, education level and family income level with some factors on PA were significant (assessed by the statistical significance of interaction terms) and literature suggests that determinants or correlates for PA may differ by age [13], sex [11-13] and socioeconomic status [12,14], our analyses were stratified by age group (15–24, 25–39, 40–64, and ≥ 65), gender (men and women), education level (secondary or lower, college, and university) and family income level (<$40 K, $40 K-<$80 K and ≥ $80 K).

The logistic regression analyses were done using SAS 9.1 (SAS Institute Inc., Cary, NC, USA).

## Results

The overall sample had a mean value of PA level of 63.27 MET-hours (standard deviation: 64.39) with a median of 41.65 and an interquartile of 72.33. Table 1 displays PA levels in MET-hours and percent of people reporting an adequate level of PA (i.e., reporting sufficient PA) by the demographics of the study population as well as the mean values of age, intention, self-efficacy, perceived barriers, perceived health benefits, social support and facility availability. PA levels decreased among older people and a higher percent of people with higher education had adequate PA level compared to people with lower education. The PA level was lower among females than males, and more men reported sufficient PA than women. According to marital status, the activity level was lower among the widowed than among people of other marital status. Students, homemakers (no pay), the retired and the disabled had lower PA levels than people in other employment status. Those having no children younger than 15 years had a similar PA level as those having at least 1. In addition, people who rated their health as excellent had a higher PA level than those who rated their health as poor.

**Table 1:** Physical activity by demographics of the study population, Canada, 2002

**Variables**	**Mean (SD)**	**N**	Means of physical activity level (SD) (MET-hours*)	Sufficient PA** N (%)
Ages (yrs)	43.47 (15.71)			
15–19		267	76.40 (57.95)	240 (89.3)
20–29		877	75.70 (69.75)	751 (85.6)
30–39		1090	66.63 (65.96)	879 (80.6)
40–49		1135	63.71 (63.57	925 (81.5)
50–59		878	59.97 (63.99)	666 (75.9)
60–69		590	51.73 (59.48)	410 (69.5)
70–79		330	36.35 (47.50)	191 (57.9)
Gender				
Male		2313	77.14 (72.39)	1916 (82.8)
Female		2854	52.02 (54.57)	2146 (75.2)
Education level				
Elementary		258	55.22 (65.44)	160 (62.0)
Secondary		2072	70.71 (71.39)	1608 (77.6)
Tech school or college		1145	65.16 (63.77)	921 (80.4)
University or higher		1621	54.04 (52.99)	1322 (81.6)
Marital status				
Living with a partner		3033	62.52 (64.72)	2365 (78.0)
Separated or divorced		532	67.71 (69.06)	413 (77.6)
Widowed		281	39.44 (50.19)	168 (59.8)
Single not married		1298	68.73 (63.46)	1096 (84.4)
Employment status				
Work full-time		2503	70.04 (68.00)	2082 (83.2)
Work part-time		630	69.85 (65.27)	525 (83.3)
In school not working		348	57.70 (51.85)	290 (83.3)
Homemaker (no pay)		317	45.10 (47.48)	232 (73.2)
Laid off or unemployed		217	65.74 (66.30)	161 (74.2)
Disabled		160	23.98 (37.90)	77 (48.1)
Retired		676	43.44 (48.85)	443 (65.5)
Self-employed		276	85.18 (78.85)	221 (80.1)
Number of children aged 1–14 yrs				
0		3418	60.77 (63.15)	2638 (77.2)
1		595	67.49 (66.60)	474 (79.7)
2		581	62.97 (63.50)	459 (79.0)
≥ 3		218	66.77 (68.80)	173 (79.4)
Household income (Canadian $)				
<20,000		649	52.30 (60.30)	440 (67.8)
20,000 – <50,000		1684	68.55 (68.19)	1326 (78.7)
50,000 – <80,000		1084	65.97 (63.73)	903 (83.3)
≥ 80,000		962	61.12 (59.11)	805 (83.7)
Self-rated health				
Excellent		1424	72.37 (67.78)	1200 (84.3)
Good		2775	63.31 (63.56)	2214 (79.8)
Fair		742	52.69 (59.37)	528 (71.2)
Poor		204	39.01 (57.37)	103 (50.5)
Intention	5.94 (0.92)			
Self-efficacy (mean score)	5.67 (1.26)			
Perceived barriers to PA (mean score)	3.85 (1.41)			
Perceived health benefit (mean score)	5.83 (0.68)			
Social support (mean score)	4.11 (1.08)			
Facility availability (mean score)	2.25 (0.45)			

*The calculation of MET-hours shown in the methods section.

**Sufficient PA was defined in the methods section.

The logistic regression result of the effects of various factors on PA, overall and by sex is shown in Table 2. Both men and women who had a poor health status were less likely to have sufficient PA than those who reported good health status. Higher education level was associated with a statistically significantly higher chance of having sufficient PA for the total sample and for women, but the association was not significant for men. People with higher family income level had increased odds of having sufficient PA for both sexes. Intention, self-efficacy and perceived health benefits were all positively associated with PA for the total sample and for both sexes. Perceived barriers were negatively associated with PA, but the association was not significant among men. Social support was not significantly associated with the odds of having sufficient PA for both genders. Although the unadjusted odd ratios (ORs) suggested that PA facility availability was significantly associated with PA, the multivariate-adjusted ORs were not significant for both men and women.

**Table 2:** Individual, social, and physical environmental correlates of sufficient PA among Canadians, overall and by gender, 2002

**Variable**	**Men and Women**		**Women**		**Men**	
			
	**Crude**	**Adjusted**	**Crude**	**Adjusted**	**Crude**	**Adjusted**
	**OR (95% CI)**	OR (95% CI) † *	**OR (95% CI)**	**OR (95% CI) †**	**OR (95% CI)**	**OR (95% CI) †**
Self-rated health						
Excellent	1.00 (reference)	1.00 (reference)	1.00 (reference)	1.00 (reference)	1.00 (reference)	1.00 (reference)
Good	0.80 (0.66–0.96)	0.96 (0.78–1.17)	0.80 (0.63–1.01)	1.04 (0.80–1.33)	0.75 (0.54–1.03)	0.85 (0.60–1.19)
Fair	0.43 (0.34–0.54)	0.83 (0.64–1.07)	0.43 (0.32–0.57)	0.95 (0.68–1.32)	0.41 (0.28–0.60)	0.68 (0.45–1.03)
Poor	0.19 (0.14–0.26)	0.42 (0.29–0.61)	0.23 (0.16–0.35)	0.60 (0.37–0.98)	0.13 (0.08–0.22)	0.25 (0.14–0.43)
Education						
Elementary	1.00 (reference)	1.00 (reference)	1.00 (reference)	1.00 (reference)	1.00 (reference)	1.00 (reference)
Secondary	2.58 (1.96–3.41)	1.69 (1.24–2.32)	3.12 (2.17–4.47)	2.15 (1.44–3.23)	1.97 (1.25–3.11)	1.19 (0.71–2.00)
Tech school or college	3.21 (2.37–4.34)	1.75 (1.24–2.46)	3.99 (2.70–5.88)	2.18 (1.41–3.39)	2.45 (1.49–4.05)	1.25 (0.70–2.22)
University	3.63 (2.71–4.85)	1.85 (1.32–2.59)	4.92 (3.36–7.19)	2.43 (1.57–3.75)	2.38 (1.48–3.83)	1.22 (0.70–2.12)
Family income						
<$20 K	1.00 (reference)	1.00 (reference)	1.00 (reference)	1.00 (reference)	1.00 (reference)	1.00 (reference)
$20–<$50 K	1.92 (1.55–2.38)	1.37 (1.07–1.74)	1.87 (1.44–2.44)	1.36 (1.01–1.82)	1.86 (1.28–2.70)	1.37 (0.89–2.11)
$50–<$80 K	2.84 (2.21–3.64)	1.62 (1.22–2.15)	2.69 (1.96–3.70)	1.59 (1.12–2.27)	2.74 (1.80–4.15)	1.68 (1.04–2.71)
≥ $80 K	3.05 (2.35–3.97)	1.69 (1.25–2.29)	2.66 (1.90–3.73)	1.49 (1.01–2.19)	3.16 (2.05–4.87)	1.98 (1.19–3.27)
Intention						
Low intention	1.00 (reference)	1.00 (reference)	1.00 (reference)	1.00 (reference)	1.00 (reference)	1.00 (reference)
Moderate intention	2.22 (1.86–2.65)	1.66 (1.37–2.02)	2.23 (1.78–2.79)	1.73 (1.35–2.22)	2.17 (1.62–2.90)	1.57 (1.14–2.15)
Fully intentional	4.14 (3.25–5.27)	2.26 (1.73–2.95)	4.68 (3.41–6.41)	2.59 (1.83–3.67)	3.38 (2.31–4.95)	1.84 (1.21–2.79)
Intention as continuous	2.06 (1.83–2.33)	1.38 (1.27–1.50)	2.17 (1.86–2.54)	1.40 (1.25–1.56)	1.87 (1.54–2.28)	1.36 (1.21–1.54)
Self-efficacy (mean score)	1.69 (1.60–1.78)	1.50 (1.41–1.59)	1.66 (1.54–1.78)	1.51 (1.40–1.63)	1.70 (1.56–1.85)	1.48 (1.34–1.62)
Perceived health benefit (mean score)	1.40 (1.27–1.54)	1.17 (1.05–1.31)	1.39 (1.22–1.58)	1.15 (1.00–1.34)	1.49 (1.29–1.73)	1.22 (1.03–1.45)
Perceived barriers to PA (mean score)	0.86 (0.81–0.90)	0.94 (0.88–0.99)	0.82 (0.77–0.88)	0.91 (0.84–0.99)	0.91 (0.84–0.98)	0.97 (0.88–1.06)
Social support (mean score)	1.03 (0.97–1.11)	1.02 (0.94–1.11)	1.01 (0.93–1.10)	1.01 (0.91–1.12)	1.10 (0.99–1.23)	1.06 (0.94–1.21)
Facility availability (mean score)	1.56 (1.33–1.83)	1.12 (0.94–1.34)	1.45 (1.18–1.78)	1.10 (0.87–1.37)	1.62 (1.25–2.11)	1.22 (0.91–1.62)

† OR adjusted for age, self-rated health, education, intention, self-efficacy, perceived health benefit, perceived barriers and facility availability.

Analyses for family income were not adjusted for education.

* OR also adjusted for sex

Stratified analyses by age group (15–24, 25–39, 40–64 and 65–79 years) (Table 3) show that self-efficacy was positively and self-rated health was negatively associated with PA for people in all 4 age groups. Higher education level was associated with higher odds of reporting sufficient PA for people in the two older groups, and higher family income level was associated with increased ORs of having sufficient PA for people in the 3 older groups, whereas the association with intention was significant for people in the 3 younger groups only. While perceived barriers were associated with lower ORs of having sufficient PA for the 3 younger groups, the association with perceived health benefits was significant only for the two older groups. Social support and facility availability were not significantly associated with multivariate adjusted ORs of having sufficient PA for any age group.

**Table 3:** Individual, social and physical environmental correlates of physical activity among Canadians, by age group, 2002

**Variable**	**15–24 yrs ** **(n = 645)**		**25–39 yrs ** **(n = 1589)**		**40–64 yrs ** **(n = 2296)**		**65–79 yrs ** **(n = 637)**	
				
	**Crude**	**Adjusted**	**Crude**	**Adjusted**	**Crude**	**Adjusted**	**Crude**	**Adjusted**
	**OR ** **(95% CI)**	**OR ** **(95% CI)†**	**OR ** **(95%CI)**	**OR ** **(95%CI) †**	**OR ** **(95% CI)**	**OR ** **(95% CI) †**	**OR ** **(95% CI)**	**OR ** **(95% CI) †**
Self-rated health								
Excellent	1.00 (reference)	1.00 (reference)	1.00 (reference)	1.00 (reference)	1.00 (reference)	1.00 (reference)	1.00 (reference)	1.00 (reference)
Good	1.14 (0.50–2.57)	1.76 (0.73–4.27)	0.83 (0.59–1.15)	0.98 (0.69–1.39)	0.76 (0.58–1.00)	0.91 (0.68–1.22)	0.78 (0.48–1.25)	0.80 (0.47–1.37)
Fair	0.41 (0.17–1.01)	0.69 (0.26–1.87)	0.71 (0.43–1.17)	0.99 (0.57–1.70)	0.39 (0.28–0.54)	0.76 (0.53–1.10)	0.47 (0.28–0.81)	0.70 (0.38–1.31)
Poor	0.33 (0.06–1.72)	0.63 (0.10–3.79)	0.23 (0.12–0.44)	0.38 (0.18–0.79)	0.21 (0.13–0.32)	0.42 (0.25–0.70)	0.18 (0.08–0.37)	0.27 (0.11–0.64)
Education								
Elementary	1.00 (reference)	1.00 (reference)	1.00 (reference)	1.00 (reference)	1.00 (reference)	1.00 (reference)	1.00 (reference)	1.00 (reference)
Secondary	0.94 (0.12–7.43)	0.41 (0.04–4.67)	1.95 (0.80–4.74)	1.12 (0.41–3.07)	2.54 (1.69–3.83)	2.48 (1.58–3.91)	1.47 (0.92–2.35)	1.43 (0.85–2.42)
Tech school or college	0.90 (0.11–7.62)	0.35 (0.03–4.16)	1.99 (0.82–4.87)	1.10 (0.40–3.05)	3.09 (1.99 (4.82)	2.64 (1.62–4.32)	2.59 (1.34–5.00)	2.38 (1.16–4.91)
University	1.87 (0.20–17.2)	0.84 (0.07–11.0)	1.91 (0.79–4.60)	1.03 (0.37–2.84)	4.22 (2.74–6.50)	3.13 (1.93–5.08)	2.29 (1.29–4.05)	1.67 (0.87–3.21)
Family income								
<$20 K	1.00 (reference)	1.00 (reference)	1.00 (reference)	1.00 (reference)	1.00 (reference)	1.00 (reference)	1.00 (reference)	1.00 (reference)
$20–<$50 K	1.36 (0.58–3.20)	1.14 (0.45–2.86)	2.18 (1.39–3.42)	1.78 (1.08–2.93)	2.36 (1.69–3.32)	1.63 (1.11–2.38)	1.25 (0.82–1.91)	0.89 (0.54–1.46)
$50–<$80 K	1.11 (0.42–2.91)	0.75 (0.26–2.14)	2.66 (1.64–4.32)	1.90 (1.12–3.25)	2.91 (2.01–4.22)	1.85 (1.22–2.82)	7.62 (2.61–22.3)	5.18 (1.58–17.0)
≥ $80 K	-	-	2.28 (1.38–3.74)	1.56 (0.89–2.74)	3.50 (2.39–5.13)	2.17 (1.39–3.39)	3.98 (1.32–12.0)	2.28 (0.67–7.69)
Intention								
Low intention	1.00 (reference)	1.00 (reference)	1.00 (reference)	1.00 (reference)	1.00 (reference)	1.00 (reference)	1.00 (reference)	1.00 (reference)
Moderate intention	4.53 (2.29–8.98)	2.92 (1.39–6.12)	2.14 (1.51–3.04)	1.76 (1.21–2.54)	2.46 (1.91–3.17)	1.70 (1.29–2.23)	1.49 (0.96–2.33)	1.17 (0.71–1.94)
Fully intentional	5.64 (2.03–15.6)	2.80 (0.94–8.36)	4.22 (2.60–6.85)	2.73 (1.63–4.57)	4.84 (3.41–6.88)	2.39 (1.64–3.50)	2.64 (1.50–4.66)	1.34 (0.71–2.55)
Intention as continuous	1.91 (1.47–2.49)	1.69 (1.25–2.29)	1.81 (1.55–2.11)	1.61 (1.37–1.91)	1.71 (1.54–1.89)	1.37 (1.22–1.53)	1.39 (1.18–1.65)	1.14 (0.95–1.38)
Self-efficacy (mean score)	1.73 (1.37–2.19)	1.58 (1.20–2.07)	1.66 (1.50–1.85)	1.56 (1.39–1.74)	1.67 (1.54–1.81)	1.47 (1.35–1.60)	1.63 (1.44–1.85)	1.49 (1.30–1.71)
Perceived barriers to PA (mean score)	0.71 (0.54–0.92)	0.71 (0.52–0.97)	0.88 (0.79–0.97)	0.94 (0.84–1.06)	0.83 (0.77–0.90)	0.92 (0.84–1.00)	0.93 (0.83–1.04)	1.07 (0.92–1.23)
Perceived health benefit (mean score)	0.97 (0.60–1.54)	0.86 (0.52–1.41)	1.25 (1.01–1.54)	1.14 (0.90–1.44)	1.52 (1.32–1.74)	1.23 (1.06–1.43)	1.57 (1.23–2.01)	1.34 (1.02–1.76)
Social support (mean score)	1.01 (0.73–1.39)	1.02 (0.69–1.50)	0.96 (0.83–1.10)	0.96 (0.82–1.13)	1.03 (0.94–1.14)	1.11 (0.99–1.24)	0.97 (0.84–1.12)	0.97 (0.81–1.16)
Facility availability (mean score)	1.55 (0.74–3.27)	1.07 (0.47–2.47)	1.34 (0.97–1.84)	1.12 (0.80–1.58)	1.62 (1.29–2.03)	1.23 (0.96–1.58)	1.21 (0.84–1.76)	0.94 (0.61–1.44)

† OR adjusted for age, self-rated health, education, intention, self-efficacy, perceived health benefit, perceived barriers and facility availability.

Analyses for family income were not adjusted for education.

With the stratified analyses by education level (secondary or lower, technical school or college, and university) (Table 4), we found that intention and self-efficacy were positively, and self-rated health were negatively, associated with PA for all 3 groups. Perceived barriers were associated with lower odds of having sufficient PA for all 3 groups, although the associations were not significant. Facility availability was significantly associated with higher odds of having sufficient PA among people with a university degree; however, social support was not significantly associated with PA for all 3 groups. The stratified analyses by family income level showed similar results as by education level (Table 5), except there were no independent effects of perceived barriers and facility availability on PA among people with a family income higher than $80,000.

**Table 4:** Individual, social and physical environmental correlates of sufficient PA among Canadians, by education level, 2002

**Variable**	**Secondary or lower**		**Tech school or college**		**University**	
			
	**Crude**	**Adjusted**	**Crude**	**Adjusted**	**Crude**	**Adjusted**
	**OR (95% CI)**	**OR (95% CI) †**	**OR (95% CI)**	**OR (95% CI) †**	**OR (95% CI)**	**OR (95% CI) †**
Self-rated health						
Excellent	1.00 (reference)	1.00 (reference)	1.00 (reference)	1.00 (reference)	1.00 (reference)	1.00 (reference)
Good	1.02 (0.77–1.34)	1.14 (0.84–1.52)	0.66 (0.43–1.01)	0.77 (0.49–1.21)	0.67 (0.48–0.93)	0.89 (0.62–1.28)
Fair	0.51 (0.37–0.70)	0.84 (0.60–1.19)	0.42 (0.25–0.72)	0.76 (0.42–1.37)	0.48 (0.29–0.79)	0.94 (0.54–1.66)
Poor	0.24 (0.16–0.37	0.49 (0.30–0.79)	0.16 (0.08–0.33)	0.39 (0.17–0.89)	0.14 (0.07–0.29)	0.24 (0.11–0.53)
Intention						
Low intention	1.00 (reference)	1.00 (reference)	1.00 (reference)	1.00 (reference)	1.00 (reference)	1.00 (reference)
Moderate intention	2.13 (1.68–2.72)	1.63 (1.25–2.13)	2.84 (1.93–4.18)	2.22 (1.47–3.35)	1.97 (1.37–2.84)	1.35 (0.90–2.03)
Fully intentional	3.99 (2.81–5.66)	2.42 (1.65–3.55)	4.71 (2.81–7.89)	2.64 (1.52–4.59)	3.57 (2.24–5.68)	1.71 (1.02–2.86)
Intention as continuous	1.61 (1.46–1.77)	1.39 (1.25–1.55)	1.78 (1.52–2.09)	1.49 (1.26–1.78)	1.66 (1.42–1.94)	1.27 (1.07–1.52)
Self-efficacy (mean score)	1.61 (1.49–1.73)	1.40 (1.29–1.52)	1.75 (1.54–1.98)	1.54 (1.35–1.76)	1.81 (1.63–2.01)	1.68 (1.50–1.88)
Perceived barriers to PA (mean score)	0.92 (0.85–0.98)	0.96 (0.88–1.04)	0.76 (0.69–0.88)	0.88 (0.76–1.01)	0.86 (0.77–0.96)	0.96 (0.84–1.09)
Perceived health benefit (mean score)	1.35 (1.20–1.53)	1.17 (1.02–1.35)	1.25 (0.98–1.58)	1.10 (0.85–1.42)	1.63 (1.31–2.04)	1.39 (1.09–1.77)
Social support (mean score)	1.11 (1.01–1.22)	1.05 (0.95–1.17)	0.86 (0.73–1.01)	0.89 (0.74–1.07)	1.13 (0.98–1.29)	1.08 (0.93–1.27)
Facility availability (mean score)	1.42 (1.14–1.76)	1.05 (0.83–1.33)	1.21 (0.83–1.76)	1.01 (0.67–1.52)	1.84 (1.32–2.55)	1.50 (1.05–2.14)

† OR adjusted for age, gender, self-rated health, intention, self-efficacy, perceived health benefit, perceived barriers and facility availability.

**Table 5:** Individual, social and physical environmental correlates of sufficient PA among Canadians, by family income, 2002

**Variable**	**< $40 K**		**$40 K – <$80 K**		**≥ $80 K**	
			
	**Crude**	**Adjusted**	**Crude**	**Adjusted**	**Crude**	**Adjusted**
	**OR (95% CI)**	**OR (95% CI) †**	**OR (95% CI)**	**OR (95% CI) †**	**OR (95% CI)**	**OR (95% CI) †**
Self-rated health						
Excellent	1.00 (reference)	1.00 (reference)	1.00 (reference)	1.00 (reference)	1.00 (reference)	1.00 (reference)
Good	0.81 (0.59–1.11)	0.99 (0.70–1.38)	1.00 (0.71–1.42)	1.24 (0.86–1.78)	0.62 (0.38–1.00)	0.68 (0.41–1.14)
Fair	0.44 (0.31–0.62)	0.82 (0.55–1.21)	0.72 (0.45–1.16)	1.40 (0.82–2.39)	0.22 (0.12–0.43)	0.30 (0.15–0.61)
Poor	0.19 (0.12–0.30)	0.51 (0.30–0.86)	0.18 (0.09–0.33)	0.31 (0.15–0.62)	0.21 (0.08–0.58)	0.22 (0.07–0.68)
Intention						
Low intention	1.00 (reference)	1.00 (reference)	1.00 (reference)	1.00 (reference)	1.00 (reference)	1.00 (reference)
Moderate intention	2.15 (1.64–2.82)	1.67 (1.23–2.27)	1.86 (1.31–2.62)	1.31 (0.90–1.90)	2.86 (1.76–4.64)	2.38 (1.42–3.99)
Fully intentional	3.30 (2.27–4.80)	1.86 (1.23–2.82)	4.99 (2.97–8.39)	2.76 (1.59–4.80)	6.69 (3.40–13.17)	4.09 (2.00–8.38)
Intention as continuous	1.60 (1.44–1.78)	1.34 (1.19–1.51)	1.62 (1.39–1.88)	1.31 (1.11–1.54)	1.93 (1.57–2.37)	1.72 (1.38–2.15)
Self-efficacy (mean score)	1.74 (1.60–1.90)	1.55 (1.41–1.71)	1.76 (1.58–1.97)	1.60 (1.42–1.80)	1.61 (1.40–1.85)	1.43 (1.23–1.66)
Perceived barriers to PA (mean score)	0.89 (0.82–0.96)	0.95 (0.87–1.04)	0.88 (0.79–0.98)	0.96 (0.85–1.08)	0.97 (0.83–1.13)	1.12 (0.93–1.35)
Perceived health benefit (mean score)	1.37 (1.19–1.57)	1.18 (1.00–1.39)	1.44 (1.20–1.72)	1.19 (0.97–1.45)	1.46 (1.05–2.03)	1.52 (1.05–2.20)
Social support (mean score)	1.01 (0.92–1.12)	0.90 (0.81–1.04)	1.09 (0.94–1.26)	1.06 (0.90–1.24)	1.17 (0.97–1.40)	1.16 (0.94–1.43)
Facility availability (mean score)	1.38 (1.08–1.77)	1.08 (0.81–1.42)	1.56 (1.12–2.18)	1.21 (0.85–1.72)	1.26 (0.82–1.93)	1.07 (0.67–1.70)

† OR adjusted for age, gender, self-rated health, intention, self-efficacy, perceived health benefit, perceived barriers and facility availability.

## Discussion

Our study results showed that education, family income, self-rated health, intention, self-efficacy, perceived barriers, perceived health benefits, and facility availability were independently related to PA. Self-efficacy and intention were the strongest correlates and had the greatest effect on PA. Self-rated health, family income, perceived health benefits, and perceived barriers were also consistently associated with PA. In addition, the effects of perceived health benefits, education and family income were more salient to older people, whereas the influence of education was more important to women and the influence of perceived barriers was more salient to women and younger people. Furthermore, facility availability was more strongly associated with PA among people with a university degree than people with a lower education level.

This study found that higher self-efficacy was consistently related to higher PA across gender, age group, education level and family income level. This finding is in agreement with other studies on an array of populations [11,13,16-22]. Confidence in personal ability to carry out a behaviour (i.e., self-efficacy) plays a central role in behaviour change and influences the direction, intensity and persistence of the behaviour [23]. This focal belief is the foundation of human motivation and action. People who have higher PA self-efficacy will perceive fewer barriers to PA or be less influenced by them, be more likely to pursue perceived benefits of being physically active, and be more likely to enjoy PA [23]. One study [24] suggests that self-efficacy determines whether people translate perceived risk into a search for health information and whether they translate their knowledge into healthy behavioural action. The study [24] also found that knowledge-behaviour correlations were greater among those with high self-efficacy, increased among those who raised their self-efficacy, and decreased among those who reduced their self-efficacy. Intervention studies also showed that enhancement or manipulation of perceived self-efficacy resulted in an increase in PA level or in adherence and maintenance of the exercise behaviour [16,25-27]. On the other hand, engagement in PA can affect a person's self-efficacy [20]. A prospective study found that participants who exercised more frequently during a 6-month structured program had a more positive exercise experience, which, in turn, enhanced their self-efficacy at program end, resulting in higher levels of exercise participation at 6- and 18-month follow-up [19]. Self-efficacy is usually higher among men than among women and is positively related to socioeconomic status [28].

Intention is another important independent correlate for PA in this study. Our finding of the positive influence of intention on PA participation corroborates those of other studies [29-32]. Intention, an essentially proximal goal, would provide self-incentives and guides for health habits as well as help people to succeed by enlisting effort and guiding action [23].

The strong effects of self-efficacy and intention on PA suggest that interventions designed to increase PA should target self-efficacy and intention. Self-efficacy can be influenced by reinforcement history, observational learning, and perceived exertion [33]. Therefore, future research is needed to identify how those influences can be optimally incorporated into interventions that will increase people's beliefs on their ability and motivation/intention to be physically active.

Our results indicate that higher SES, including higher family income level and education level, is positively associated with PA, although the association between education and PA was significant among women but not among men. Many studies found a positive association between higher education or higher income and PA levels [6,33-35]. People with higher education levels are more likely to have better general health, higher self-efficacy (due to stronger problem-solving and coping capacities arising from educational experience), more social support, and a greater capacity to seek, understand, and act on health messages that promote PA [6,14,36,37]. In addition, people with higher family income levels usually have better health (due in part to better access to health care resources), have better access to PA facilities and opportunities, can choose and afford to live in a pleasant and activity-friendly environment, and have less barriers to PA [6,14]. Our results of stronger associations with self-efficacy, perceived health benefits and facility availability among people with a university degree than among those with lower educations, as well as stronger associations with intention and perceived health benefits and a weaker/no association with perceived barriers among people in the category of highest family income level provide supports to the role of the SES on PA level. No significant association between education and PA among men could be because that men with lower education levels are more likely to have jobs of physical labour (therefore have higher occupational PA) than people with higher education levels [38]; therefore, men with lower education levels may have similar total PA as those with higher education levels even though they may have lower recreational PA. It is also possible that, compared to men with lower education levels, women with lower education levels are more likely to have sedentary jobs such as clerks and secretaries [38], therefore having an occupational PA level more similar to women with higher education levels.

This study also found that self-rated health was strongly and consistently related to PA across sex, age group and SES. Perceived poor health has been reported to be associated with lower PA level in other studies [22,34,39,40]. A cross-sectional study of 16,230 respondents in the 15 member states of the European Union also observed a higher level of total PA associated with better self-related health across populations [41]. However, one study of urban women indicated that self-rated health was not a significant correlate of leisure-time PA [42].

Our finding of the positive correlation between perceived health benefits and PA level is in line with other studies [11,13,18,30]. The results of two studies on samples of mainly males [21,22] also support our finding. One possible explanation for the more salient effect of PA's health benefits on PA level in older people than in younger people is that older people usually have more health problems, therefore consider PA health benefits more important for them than younger people do. Younger people might consider other benefits more important in their decision to participate in PA, such as enjoyment, social interaction, improvement of self-esteem, better shape, increased attractiveness, and strength. Therefore, future studies assessing PA benefits should include not only health benefits but also other psychological benefits, while there is a need to include both benefits in the education message in developing interventions of promoting PA.

Perceived barriers as an important factor for PA participation have been demonstrated in many studies [12,13,32,43]. That perceived personal barriers appear to be more important to women than to men might reflect the situation that women devote more of their time to their multiple responsibilities as workers, housekeepers, mothers and wives. The greater effect of barriers on PA in women than in men may be also because men may enjoy PA more than women, and men usually have higher self-efficacy for PA thus perceiving less barriers or less influenced by barriers [28]. In addition, on average, men have higher levels of occupational PA than women [38] while perceived barriers to PA are mainly related to recreational PA. One possible reason of a stronger effect of perceived barriers among people <65 years than among seniors might be that seniors are usually retired so they usually have more free time than younger people.

Both perceived and objectively measured physical environment factors were found to be positively related to PA level [7,9-12,18,34,44]. Availability, accessibility, convenience of destinations and facilities as well as the general functionality of the neighbourhood (e.g., traffic condition, street lighting at night, unattended dogs and safety from crime) and aesthetics have been shown to be positively associated with PA level [7,9]. A meta-analysis [10] found a modest, yet significant association between the perceived physical environment and PA. Literature suggests that the built environment can affect people's decision for participating in PA by providing cues and opportunities for activities to occur [9,45]. Some studies indicated that the physical environment also had an indirect effect on PA through self-efficacy [18,46,47]. However, our study suggests that perceived facility availability was significantly associated with PA only among people with a university degree. This study assessed only availability, while accessible, convenient and safe facilities for PA might be more strongly associated with PA than availability [48] because people would not use those available facilities if they are too expensive, not convenient and not safe. Therefore, accessibility, convenience and safety of PA facilities should be assessed in future research on the physical environment. Also, both perceived and objectively measured physical environment data should be included in the same studies.

Although we did not observe an independent effect of social support on PA, many studies have shown the importance of social support in promoting PA [11,12,17,21,22,47]. Some studies have also shown that social support has an indirect effect on PA through self-efficacy [18,19,47,49].

Limitations of our study should be considered when interpreting the results. First of all, our study was a cross-sectional design and causal inferences cannot be made because of the inability to determine temporal sequence. Prospective study designs should be considered in further research on these relationships in order to provide more insight on the question of the causal direction. Secondly, the response rate was low (51%) and there might be inherent differences between people who agreed to participate in the study and those who did not. However, earlier analyses showed no response rate bias [50]. Furthermore, this response rate was similar to that of other PA surveys in other countries such as Australia, the US and the Netherlands [11,16,17,19,42]. Another limitation common to population surveys was that PA measures were self-reported, where respondents may over-report their occasions or time spent in PA. The survey assessed total physical activity only, therefore, we could not examine the correlates for occupational, recreational and transport-related PA separately, whereas some factors are related to recreational and transport-related PA only. Future research should collect separate information on these types of PA in order to understand the differential effect of various factors on specific types of PA. Also, our current analysis did not assess mediation between different factors (individual factors such as self-efficacy and intention may mediate the influences of social factors, environmental factors and other personal factors on PA level); therefore we could not examine the indirect effects of environment variables on PA and potential pathways between variables and PA.

## Conclusion

This study identified several significant factors that were associated with PA participation. We also found differences between genders, age groups and SES in various correlates of PA. Our findings highlight the need that health promotion programs should be targeting on enhancement of people's confidence and motivation, education on health benefits of PA and reduction of barriers to achieve desired changes. Our findings also imply that interventions to promote PA need to address different factors for men and women as well as for differing socio-economic and demographic groups.

## Competing interests

The authors declare that they have no competing interests.

## Authors' contributions

SYP conceived the study, performed the analyses, wrote the manuscript and incorporated input from all other authors on the manuscript. CC and CLC conceived the socio-ecological elements and methods instrumental to the study and provided critical comments on the manuscript. MD and HM directed the overall study and provided critical comments on the manuscript. XJ helped part of the literature review. All authors have read and approved the final version of the manuscript.

## Pre-publication history

The pre-publication history for this paper can be accessed here:


